# New Disulfiram Derivatives as MAGL-Selective Inhibitors

**DOI:** 10.3390/molecules26113296

**Published:** 2021-05-30

**Authors:** Ziad Omran

**Affiliations:** Department of Pharmaceutical Sciences, Pharmacy Program, Batterjee Medical College, Jeddah 21442, Saudi Arabia; ziad.omran@bmc.edu.sa

**Keywords:** monoacylglycerol lipase, fatty acid amide hydrolase, disulfiram

## Abstract

Monoacylglycerol lipase (MAGL) is a key enzyme in the human endocannabinoid system. It is also the main enzyme responsible for the conversion of 2-arachidonoyl glycerol (2-AG) to arachidonic acid (AA), a precursor of prostaglandin synthesis. The inhibition of MAGL activity would be beneficial for the treatment of a wide range of diseases, such as inflammation, neurodegeneration, metabolic disorders and cancer. Here, the author reports the pharmacological evaluation of new disulfiram derivatives as potent inhibitors of MAGL. These analogues displayed high inhibition selectivity over fatty acid amide hydrolase (FAAH), another endocannabinoid-hydrolyzing enzyme. In particular, compound **2i** inhibited MAGL in the low micromolar range. However, it did not show any inhibitory activity against FAAH.

## 1. Introduction

Monoacylglycerol lipase (MAGL) is a serine hydrolase ~33 kDa in size that catalyzes the hydrolysis of monoglycerides (MAGs) into glycerol and free fatty acids [1]. Its substrates include MAGs of different fatty acid chain lengths and degrees of saturation (e.g., 2-arachidonoyl glycerol, 2-oleylglycerol, 2-palmitoylglycerol, and 2-stearoylglycerol), but 2-arachidonoyl glycerol (2-AG) is of particular pharmacological importance because it is one of the most abundant endocannabinoids capable of activating both the CB_1_R and CB_2_R types of cannabinoid receptors [2]. Thus, 2-AG has an important role in the regulation of pain sensations [3], addiction [4], neuroprotection [5] and even food intake [6]. Conversely, arachidonic acid (AA), the main product of MAGL action on 2-AG, is a key precursor for the synthesis of proinflammatory prostaglandins. Consequently, the inhibition of MAGL will enhance endocannabinoid signaling and reduce eicosanoid production [7].

The endocannabinoid system is also associated with metabolic disorders. For example, *Magl*^−/−^ mice showed reductions in bodyweight and serum lipid levels compared to their wild-type counterparts [8]. MAGL inhibitors also prevented glucose-stimulated and depolarization-induced insulin secretion [9]. Therefore, MAGL inhibition also represents a plausible strategy for the treatment of metabolic disorders [2].

MAGL also plays a pathophysiological role in aggressive cancers [10], showing overexpression in aggressive human cancer cells and primary tumors ranging from prostate cancer [11] to colorectal cancer [12], hepatocellular carcinoma [13] and nasopharyngeal carcinoma [13]. MAGL is thought to function through its regulation of an oncogenic signaling network of lipids by supplying a pool of free fatty acids (FFAs) that promote the migration, invasion and survival of cancer cells. Consequently, reducing the FFA levels by inhibiting MAGL also decreases cancer aggressiveness, independently of endocannabinoid signaling. These reductions in FFA levels by MGAL inhibition are only observed in aggressive cancers, because in healthy tissues MAGL controls the MAG levels but not the FFA levels. The levels of known oncogenic lysophospholipids, such as phosphatidic acid and prostaglandin (PGE2), are also significantly reduced if MAGL is inhibited. As an added benefit, MAGL inhibition also has a positive impact on cancer-associated symptoms, including pain and nausea [14].

Taken together, the available evidence supports MAGL inhibition as a new therapeutic avenue for the treatment of inflammation, neurodegeneration, metabolic disorders and cancers. In this context, a potent and highly selective MAGL inhibitor, ABX-1431, is currently undergoing phase-II clinical trials to test its efficacy in treating two neurological disorders, namely Tourette syndrome and motor tic disorder [2]. Disulfiram, an aldehyde dehydrogenase inhibitor approved by the FDA for the treatment of chronic alcoholism, is another drug that can inhibit MAGL activity [15]. Disulfiram was shown to irreversibly inhibit MAGL by the carbamylation of Cys208 and Cys242 [16], which are located in the vicinity of the MAGL active site [17]. In addition to its inhibition of MAGL, disulfiram inhibits fatty acid amide hydrolase (FAAH) [16], an enzyme responsible for the hydrolysis of both 2-AG and *N*-arachidonoylethanolamine [18]. However, because MAGL is the main enzyme responsible for 2-AG in catabolism in the brain [19,20], the aim of the present study was to develop disulfiram analogues that can selectively inhibit MAGL activity. In order to elucidate the structure–activity relationship of disulfiram derivatives, the *N*-ethyl groups of disulfiram were replaced so as to modify the bulkiness, hydrophobicity and hydrophilicity, as described below.

## 2. Results and Discussion

**Figure 1:**
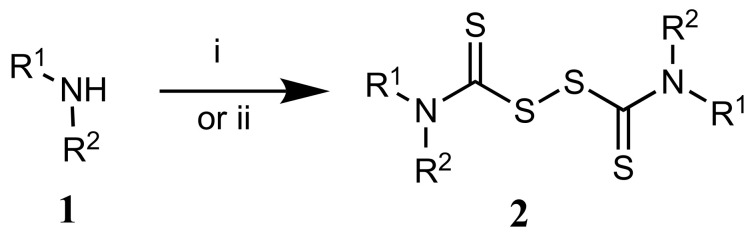
Chemical synthesis of disulfiram analogue **2**. i: CS_2_, CB_4_, DMF, RT. ii: NaOH, CS_2_, NaNO_2_, water, 0 °C.

Thiuram disulfide **2** was obtained as previously reported (Figure 1) [21]. Two equivalents of the corresponding secondary amine **1** were treated with carbon disulfide, followed by treatment with either carbon tetrabromide or sodium nitrite.

The inhibitory activity of the thiuram disulfide **2** was evaluated in vitro against human MAGL (*h*MAGL) and human FAAH (*h*FAAH), as shown in Table 1. Disulfiram is a known and potent irreversible inhibitor of MAGL, and it showed an IC_50_ in the micromolar range, in agreement with the values reported in the literature [16]. Replacing the ethyl groups with an isopropyl motif (**2b**) had no significant effect on the activity. However, the introduction of a much bulkier alkyl group, such as butyl (**2c**) or isobutyl (**2d**), decreased the inhibitory activity by up to 15-fold. Introducing polar functional groups like hydroxyl (**2e**) or carboxylate (**2f**) onto the two ethyl groups of disulfiram also seemed to be beneficial for the anti-MAGL activity. The diester derivative of the latter (**2g**), which may serve as a CNS-penetrating prodrug, also showed an MAGL-inhibitory activity in the low micromolar range. Furthermore, replacing two of the four ethyl groups in disulfiram with benzyl motifs was generally well tolerated, as compound (**2h**) retained the inhibitory activity of the reference compound, disulfiram. Substituting the phenyl *para* position of (**2h**) with a small electronegative group, such as a fluoride (**2i**) or a hydroxyl (**2j**) group, had practically no effect on the inhibitory activity, whereas introducing a carboxylate functional group at the same position of (**2k**) caused a 20-fold decrease in the activity against MAGL. The esterification of the carboxylate functions of the latter compound restored the activity to that of the non-substituted derivative (**2h**). No significant change in activity was noted following the replacement of all four ethyl groups of disulfiram with benzyl groups (**2m**).

The author also investigated whether the inhibition of the MAGL manifested by the developed derivatives was irreversible and whether it was caused by the carbamylation of Cys208 and/or Cys242 [16] located close to the catalytic Ser132 [17]. This was acheived by testing the inhibitory activity of compound (**2d**) in the absence and presence dithiothreitol (10 mM). The hydrolytic activity of MAGL inhibited by (**2d**) was restored upon the addition of dithiothreitol, thereby confirming that the developed compounds inhibit MAGL via a similar mechanism to that of disulfiram.

The selectivity of the developed molecules was assessed by evaluating their in vitro inhibitory activity against FAAH. Under the assay conditions described below, disulfiram showed an inhibitory activity against FAAH attested by an IC_50_ of 36 µM, which is ten times less than that reported in the literature [16]. Interestingly, disulfiram analogues with bulkier alkyl groups (**2c**,**d**) showed no inhibition of FAAH. By contrast, the introduction of polar functional groups on the 2 ethyl groups of disulfiram (**2e**–**g**) restored the FAAH inhibitory activity at the low micromolar range. With the exception of the phenolic derivatives (**2j**), replacing the two ethyl groups in disulfiram with substituted or nonsubstituted benzyl motifs yielded compounds that were devoid of anti-FAAH activity (**2h**,**i** and **2k**,**l**). Replacing all four ethyl groups of disulfiram with benzyl groups (**2m**) also led to the complete loss of FAAH inhibitory activity.

## 3. Materials and Methods

The commercially available compounds **2b**–**d** and **2m** were purchased from BOC Sciences, Shirley, NY 11967, USA. Compounds **2e**,**f** and **2h**–**k** were obtained as previously described [21].

### 3.1. Synthesis of Compounds **2g** and **2l**

CS_2_ (2.0 mmol) was added to a solution of amine (**1**) (4 mmol) in DMF (4 mL) in an ice-water bath. The mixture was stirred for 5 min. CBr_4_ (2 mmol) was then added and the mixture was stirred at RT for a further 30 min. The mixture was poured into ice-water (40 mL) with stirring, and was then extracted with 2 × 40 mL CH_2_Cl_2_. The organic layer was dried over MgSO_4_, concentrated under vacuum, and purified by column chromatography on silica gel to give the desired product.

*Bis(N-ethoxycarbonylethylethylthiocarbamoyl)disulphide* (**2g**): Column chromatography Silica Gel, CH_2_Cl_2_-MeOH (100/0 % to 98/2 %). Yield: 34% (viscous oil). IR: 2978, 2934, 1726, 1487, 1415, 1373, 1279, 1180, 1163 cm^−1^. ^1^H-NMR (400 MHz, CDCl_3_): 1.27 (3H, bs), 1.48 (3H, bs), 2.86 (2H, bs), 3.01 (2H, bs), 4.05–4.30 (12H, bs). ^13^C-NMR (100 MHz, CDCl_3_): 11.46, 13.50, 14.26, 14.33, 31.29, 33.03, 47.94, 49.18, 52.55, 52.85, 60.94, 61.23, 170.73, 171.72, 193.19, 193.51. HR-MS (ESI^+^) *m*/*z* [M + 1] calculated: 441.1010, found: 441.1015. The spectra are attached in the online Supplementary Material.

*Bis(N-4-ethoxycarbonylbenzylethylthiocarbamoyl)disulphide* (**2l**): Column chromatography Silica Gel, CH_2_Cl_2_ (100%). Yield: 23% (viscous oil). IR: 3059, 2976, 1710, 1610, 1481, 1458, 1408 cm^−1^. ^1^H NMR (400 MHz, CDCl_3_): 1.20–1.50 (12H, m), 4.02 (4H, bs), 4.37 (4H, q, 3J = 7.2 Hz), 5.20–5.50 (4H, m), 7.30–7.55 (4H, m), 7.95–8.15 (4H, m). ^13^C-NMR (100 MHz, CDCl_3_): 11.13, 11.31, 13.44, 14.43, 47.82, 52.53, 55.58, 59.47, 61.13, 127.54, 130.15, 139.64, 140.28, 193.94, 195.63. HR-MS (ESI^+^) *m*/*z* [M + 1] calculated: 565.1323, found: 565.1321. The spectra are attached in the online Supplementary Material.

### 3.2. MAGL Enzyme Inhibition Assay

All of the tested compounds were prepared as 10 mM stock solutions in DMSO. The compounds were tested in the 10-dose IC_50_ mode, with 3-fold serial dilution at a starting concentration of 100 μM for the compounds (**2a**–**m**) and a concentration of 10 μM for the JZL184, which was used as a positive control. The assay buffer (10 mM Tris-HCl with 1 mM EDTA, pH 7.2) was used to dilute the FAAH human recombinant enzyme (Reaction Biology Corp., Malvern, 19355, PA, USA) to a final concentration of 10 nM. The substrate, 4-nitrophenylacetate, was used at a final concentration of 250 μM. The plate was mixed for 30 s and incubated at room temperature for 30 min. The plate was read at an absorption wavelength of 405 nm using a CLARIOstar plate reader to detect the release of the by-product 4-nitrophenol. The measurement of IC_50_ for compound **2d** was repeated in the presence 10 mM dithiothreitol in order to check the reversibility of the MAGL inhibition by the developed compounds.

### 3.3. FAAH Enzyme Inhibition Assay

The FAAH inhibition assay was performed using a Fatty Acid Amide Hydrolase Inhibitor Screening Assay Kit (Item # 10005196), Cayman (1180 E Ellsworth Rd Ann Arbor, MI, USA). The compounds were tested in the 10-dose IC_50_ mode, with 3-fold serial dilution at a starting concentration of 100 μM for the compounds (**2a**–**m**) and a concentration of 5 μM for JZL195, which was used as a positive control. The manufacturer’s protocol was followed to perform the assay. The assay buffer (125 mM Tri-HCl with 1 mM EDTA, pH 9.0) was used to dilute the FAAH human recombinant enzyme. AMC-Arachidonoyl amide, at a concentration of 400 μM, was then used as the FAAH substrate. The samples were mixed for 30 s and incubated at 37 °C for 30 min. The fluorescent byproduct AMC (7-amino-4-methylcoumarin) released by the FAAH enzyme was detected and quantified at an excitation wavelength of 355 nm and an emission wavelength of 460 nm using an EnVision plate reader.

## 4. Conclusions

In conclusion, MAGL has a central function in the endocannabinoid system, and MAGL inhibitors are promising therapeutic agents for various disorders, including inflammation, neurodegeneration, metabolic disorders, and even cancers. In this work, it was shown that disulfiram analogues in which the ethyl groups were replaced by bulkier hydrophobic alkyl groups or (un)substituted benzyl groups inhibited the MAGL activity by binding irreversibly to cysteine residues in the vicinity of the MAGL active site. By contrast, these derivatives were completely devoid of inhibitory activity against FAAH. The obtained results prompt us to evaluate the biological comportment of these new derivatives by further in vitro and in vivo studies.

## Figures and Tables

**Table 1:**
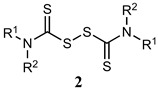
Inhibition of *h*MGAL and *h*FAAH by disulfiram and its analogues **2b**–**m**. JZL-184 and JZL-195 were included in the test as known inhibitors. The data are presented as the average of at least two different experiments ± the standard error.

Compound.	R^1^	R^2^	IC_50_ (µM) ± SE (*h*MAGL)	IC_50_ (µM) ± SE (*h*FAAH)
**2a** (Disulfiram)	Et	Et	0.95 ± 0.26	36.20 ± 10.99
**2b**	-CH(CH_3_)_2_	-CH(CH_3_)_2_	1.89 ± 0.37	NI
**2c**	-CH_2_CH_2_CH_2_CH_3_	-CH_2_CH_2_CH_2_CH_3_	7.14 ± 0.21	NI
**2d**	-CHCH_2_(CH_3_)_2_	-CHCH_2_(CH_3_)_2_	13.42 ± 4.79	NI
**2e**	Et	-CH_2_CH_2_OH	0.72 ± 0.20	25.35 ± 3.26
**2f**	Et	-CH_2_CH_2_COOH	0.87 ± 0.21	12.76 ± 1.57
**2g**	Et	-CH_2_CH_2_COOEt	3.53 ± 1.32	10.05 ± 0.68
**2h**	Et	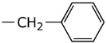	5.53 ± 2.62	NI
**2i**	Et	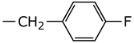	3.58 ± 1.54	NI
**2j**	Et	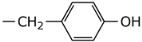	3.71 ± 1.28	38.26 ± 7.96
**2k**	Et	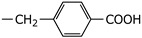	22.63 ± 16.83	NI
**2l**	Et	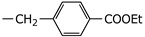	5.03 ± 2.35	NI
**2m**	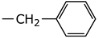	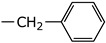	3.58 ± 0.95	NI
**JZL-184**	–		0.02 ± 0.00	–
**JZL-195**			–	0.04± 0.02

## Data Availability

Not applicable.

## Supplementary Materials

The synthesis protocols for mines **1g** and **1l**, and the IR, ^1^HNMR, ^13^CNMR and HRMS spectra for the new compounds, are included in the online supplementary material.

## Sample Availability

Samples of the compounds described in this paper are available from the author.

## References

[1] Vandevoorde S., Saha B., Mahadevan A., Razdan R.K., Pertwee R.G., Martin B.R., Fowler C.J. (2005). Influence of the degree of unsaturation of the acyl side chain upon the interaction of analogues of 1-arachidonoylglycerol with monoacylglycerol lipase and fatty acid amide hydrolase. Biochem. Biophys. Res. Commun..

[2] Deng H., Li W. (2020). Monoacylglycerol lipase inhibitors: Modulators for lipid metabolism in cancer malignancy, neurological and metabolic disorders. Acta Pharm. Sin. B.

[3] Calignano A., La Rana G., Giuffrida A., Piomelli D. (1998). Control of pain initiation by endogenous cannabinoids. Nat. Cell Biol..

[4] Maldonado R., Valverde O., Berrendero F. (2006). Involvement of the endocannabinoid system in drug addiction. Trends Neurosci..

[5] Sánchez A., García-Merino A. (2012). Neuroprotective agents: Cannabinoids. Clin. Immunol..

[6] Di Marzo V., Goparaju S.K., Wang L., Liu J., Bátkai S., Járai Z., Fezza F., Miura G.I., Palmiter R.D., Sugiura T. (2001). Leptin-regulated endocannabinoids are involved in maintaining food intake. Nat. Cell Biol..

[7] Nomura D.K., Morrison B., Blankman J.L., Long J.Z., Kinsey S.G., Marcondes M.C.G., Ward A.M., Hahn Y.K., Lichtman A.H., Conti B. (2011). Endocannabinoid Hydrolysis Generates Brain Prostaglandins That Promote Neuroinflammation. Science.

[8] Douglass J.D., Zhou Y.X., Wu A., Zadrogra J.A., Gajda A.M., Lackey A.I., Lang W., Chevalier K.M., Sutton S.W., Zhang S.-P. (2015). Global deletion of MGL in mice delays lipid absorption and alters energy homeostasis and diet-induced obesity. J. Lipid Res..

[9] Berdan C.A., Erion K.A., Burritt N.E., Corkey B.E., Deeney J.T. (2016). Inhibition of Monoacylglycerol Lipase Activity Decreases Glucose-Stimulated Insulin Secretion in INS-1 (832/13) Cells and Rat Islets. PLoS ONE.

[10] Nomura D.K., Long J.Z., Niessen S., Hoover H.S., Ng S.-W., Cravatt B.F. (2010). Monoacylglycerol Lipase Regulates a Fatty Acid Network that Promotes Cancer Pathogenesis. Cell.

[11] Nomura D.K., Lombardi D.P., Chang J.W., Niessen S., Ward A.M., Long J.Z., Hoover H.H., Cravatt B.F. (2011). Monoacylglycerol Lipase Exerts Dual Control over Endocannabinoid and Fatty Acid Pathways to Support Prostate Cancer. Chem. Biol..

[12] Ye L., Zhang B., Seviour E., Tao K.-X., Liu X.-H., Ling Y., Chen J.-Y., Wang G.-B. (2011). Monoacylglycerol lipase (MAGL) knockdown inhibits tumor cells growth in colorectal cancer. Cancer Lett..

[13] Zhang J., Liu Z., Lian Z., Liao R., Chen Y., Qin Y., Wang J., Jiang Q., Wang X., Gong J. (2016). Monoacylglycerol Lipase: A Novel Potential Therapeutic Target and Prognostic Indicator for Hepatocellular Carcinoma. Sci. Rep..

[14] Sticht M., Long J.Z., Rock E.M., Limebeer C.L., Mechoulam R., Cravatt B.F., Parker L. (2012). Inhibition of monoacylglycerol lipase attenuates vomiting in Suncus murinus and 2-arachidonoyl glycerol attenuates nausea in rats. Br. J. Pharmacol..

[15] LaBar G., Bauvois C., Muccioli G.G., Wouters J., Lambert D.M. (2007). Disulfiram is an Inhibitor of Human Purified Monoacylglycerol Lipase, the Enzyme Regulating 2-Arachidonoylglycerol Signaling. ChemBioChem.

[16] Kapanda C.N., Muccioli G.G., LaBar G., Poupaert J.H., Lambert D.M. (2009). Bis(dialkylaminethiocarbonyl)disulfides as Potent and Selective Monoglyceride Lipase Inhibitors. J. Med. Chem..

[17] Saario S.M., Salo-Ahen O.M.H., Nevalainen T., Poso A., Laitinen J.T., Järvinen T., Niemi R. (2005). Characterization of the Sulfhydryl-Sensitive Site in the Enzyme Responsible for Hydrolysis of 2-Arachidonoyl-Glycerol in Rat Cerebellar Membranes. Chem. Biol..

[18] Cravatt B.F., Giang D.K., Mayfield S.P., Boger D.L., Lerner R.A., Gilula N.B. (1996). Molecular characterization of an enzyme that degrades neuromodulatory fatty-acid amides. Nat. Cell Biol..

[19] Blankman J.L., Simon G.M., Cravatt B.F. (2007). A Comprehensive Profile of Brain Enzymes that Hydrolyze the Endocannabinoid 2-Arachidonoylglycerol. Chem. Biol..

[20] Muccioli G., Xu C., Odah E., Cudaback E., Cisneros J.A., Lambert D.M., Lopez-Rodriguez M.L., Bajjalieh S., Stella N. (2007). Identification of a Novel Endocannabinoid-Hydrolyzing Enzyme Expressed by Microglial Cells. J. Neurosci..

[21] Omran Z. (2021). Development of new disulfiram analogues as ALDH1a1-selective inhibitors. Bioorganic Med. Chem. Lett..

