# Supplementary material for: New Disulfiram Derivatives as MAGL-Selective Inhibitors
Source: Molecules. 2021 May 30;26(11):3296. doi: 10.3390/molecules26113296 (PMC8199291; doi:10.3390/molecules26113296)
Supplement: Supplementary file 1 [file molecules-26-03296-s001.zip › molecules-1220078-supplementary.pdf]

Supplementary material

New disulfiram derivatives as MAGL-selective inhibitors

Ziad Omran[1]
Department of Pharmaceutical Sciences, Pharmacy Program, Batterjee Medical College, 21442 Jeddah, Saudi Arabia

Table of Contents

Synthesis of compounds **1g**	Page 2
Synthesis of compounds **1k** and **1l**	Page 2
Analytical data for compound **2g**	Page 4
Analytical data for compound **2l**	Page 11

[1] To whom correspondence should be addressed. e-mail: ziad.omran@bmc.edu.sa

Synthesis of amine 1g:

Amine (**1g**) was obtained by amination of 3-bromopropanoate (**3**) by benzylethylamine. Debenzylation of amine (4) by reductive cleavage over palladium metal with molecular hydrogen yielded the desired amine (1g), Figure S-1.

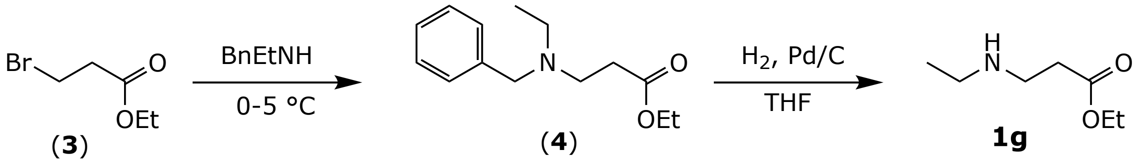

Figure S-1. Synthesis of Amine (**1g**).

Ethyl 3-(benzyl(ethyl)amino)propanoate (4):

To 2.6 mL (20 mmol) of ethyl 3-bromopropanoate (**3**) cooled to (0-5°C) was added dropwise during 5 min 6 mL (40 mmol) of benzylethylamine under vigorously stirring. After few minutes, the solution become solid. To this solid was added 30 mL of diethyl ether. The suspension was stirred vigorously for 5 min, filtered and the filtrate was concentrated in vacuo to give 3.6 g of an oil which is engaged in the next step without further purification. Yield: 76 % (light yellow oil), FT-IR: 3028, 2972, 2935, 2802, 1732, 1452, 1369, 1247, 1192, 1161, 1045, 1028 cm^{-1}. ^{1}HNMR (400 MHz, $CDCl_3$): 1.03 (3H, t, ^{3}J=7.2Hz), 1.23 (3H, t, ^{3}J=7.2Hz), 2.40-2.56 (4H, m), 3.58 (2H, s), 4.11 (2H, q, ^{3}J=7.2Hz), 7.20-7.35 (5H, m).

Ethyl 3-*N*-ethylaminopropanoate (1g):

To 2.55 g (10.8 mmol.) of ethyl 3-*N*-ethylbenzylaminopropanoate diluted in 50 mL of THF was added cautiously 1 g of palladium 10 % on carbon. The suspension was hydrogenated at atmospheric pressure and room temperature for 6 hours. The reactional medium was degassed and the suspension was filtered on a pad of celite to remove the catalyst. The filtrate is concentrated in vacuo (bath temperature at 35°C to avoid amidification) to give 1.48 g of compound (**1g**) as an oil. Yield: 94 % (light yellow oil). FT-IR: 3313, 2968, 2937, 2827, 1730, 1446, 1373, 1186, 1141, 1026 cm^{-1}. ^{1}H NMR (400 MHz, $CDCl_3$): 1.11 (3H, t, 3J=7.2Hz), 1.26 (3H, t, ^{3}J=7.2Hz), 2.52 (2H, t, $_{3}J$=6.4Hz), 2.66 (2H, q, ^{3}J=7.2Hz), 2.89 (2H, t, ^{3}J=6.4Hz), 4.14 (2H, q, ^{3}J=7.2Hz).

Synthesis of amine (1l):

Amine (**1l**) was obtained by reductive amination of commercial 4-formylbenzoic acid (**5**) using 2 equivalents of ethylamine in methanol and reducing the intermediate imine by sodium borohydride. The obtained amine (**1k**) was then esterified in refluxing ethanol to give the aimed amine (**1l**), Figure S-2.

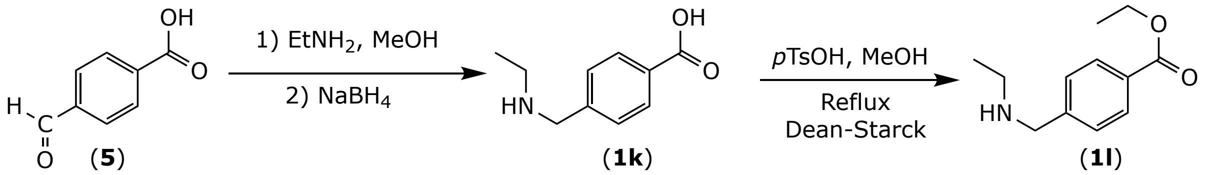

Figure S-2. Synthesis of amines (**1k** and **1l**).

4-((ethylamino)methyl)-benzoic acid (1k):

To a cold solution of 4.50 g (30 mmol) of commercial 4-formylbenzoic acid dissolved in 50 mL of dry methanol was added 20 g of molecular sieves (4A) and 67 mL of ethylamine 0.9 M in methanol. The suspension was stirred 14 hours at R.T. and filtered. To the cold filtrate was added 2.2 g (30 mmol.) of sodium borohydride by portions and the solution was stirred 15 hours. The mixture was concentrated to dryness in vacuo and cold water was added. To the aqueous layer was added HCl conc. (5 mL) to reach pH 7.15. The mixture was then concentrated to dryness under vacuum and the residual solid was extracted twice with 150 mL of methanol. The methanolic solution was dried on sodium sulphate, filtered and then concentrated to dryness under vacuum to give 4.80 g of white solid corresponding to (**1k**) (zwitterionic form). Yield: 89 % (white solid). FT-IR: 3356, 3037, 2976, 1589, 1543 cm^{-1}. 1H NMR (400 MHz, D_2O): 1.32 (3H, t, 3J=7.6Hz), 3.15 (2H, q, ^3J=7.6Hz), 4.24 (2H, s), 7.48 (2H, d, ^3J=8.4Hz), 7.85 (2H, d, ^3J=8.4Hz).

Ethyl 4-((ethylamino)methyl)-benzoate (1l):

A suspension of 2.61 g (14.56 mmol) of (**1k**) and of 3.06 g (17.77 mmol) of *p*-toluenesulfonic acid in a mixture 100 mL of dry toluene and 100 mL of ethanol was heated at reflux for 6 hours in a round bottom flask equipped with a Dean-Stark apparatus. After cooling the obtained solution, 50 mL of diethyl ether was added, and the suspension was stirred 15 min, then filtered and washed 3 times with diethyl ether. The solid was solubilized in 50 mL cold water and 25 mL. of NaOH 1N aq. was added dropwise. The aqueous solution was extracted 3 times with 10 mL of DCM. The combined organic layers are dried over Na_2SO_4 and concentrated in vacuo (bath temperature 35°C to avoid amidification) to give 0.63 g of compound (**1l**) as a light yellow oil. Yield: 20 % (light yellow oil). FT-IR: 3313, 2966, 2933, 1712, 1610, 1271, 1101, 1020, 752 cm^{-1}. ^1H NMR (400 MHz, $CDCl_3$): 1.11 (3H, t, ^3J=7.2Hz), 1.26 (3H, t, ^3J=7.2Hz), 2.52 (2H, t, ^3J=6.4Hz), 2.66 (2H, q, ^3J=7.2Hz), 2.89 (2H, t, ^3J=6.4Hz), 4.14 (2H, q, ^3J=7.2Hz).

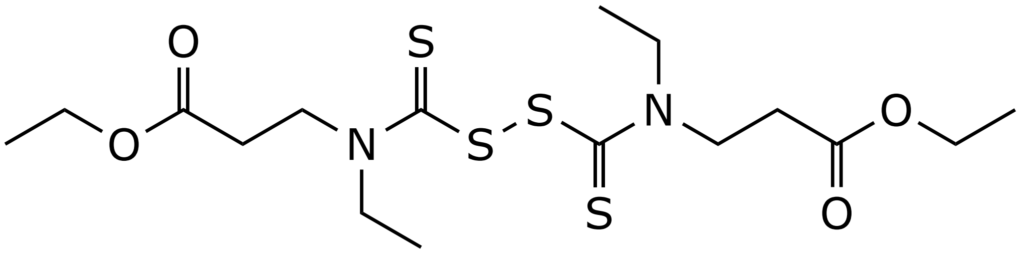

2g

Chemical Formula: $C_{16}H_{28}N_2O_4S_4$
Exact Mass: 440.0932

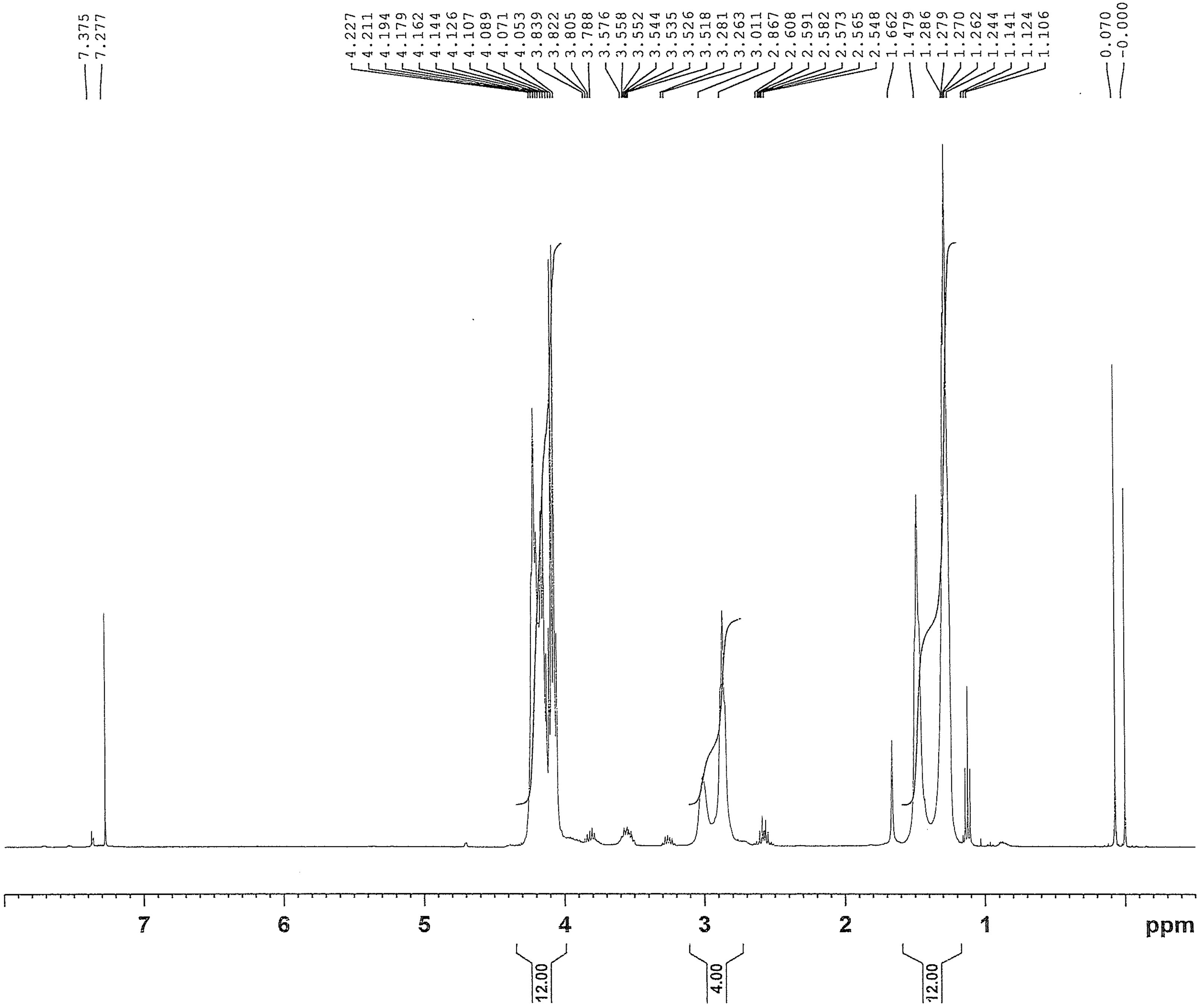

Current Data Parameters
NAME SN0321-L1
EXPNO 10
PROCNO 1

F2 - Acquisition Parameters
Date_ 20200821
Time 15.02
INSTRUM spect
PROBHD 5 mm PABBO BB/
PULPROG zg30
TD 32768
SOLVENT CDCl3
NS 16
DS 2
SWH 7978.724 Hz
FIDRES 0.243491 Hz
AQ 2.0534613 sec
RG 54.03
DW 62.667 usec
DE 6.50 usec
TE 296.2 K
D1 1.00000000 sec
TD0 1

======== CHANNEL f1 ========
SFO1 399.7524686 MHz
NUC1 1H
P1 9.00 usec
PLW1 19.20000076 W

F2 - Processing parameters
SI 65536
SF 399.7500030 MHz
WDW EM
SSB 0
LB 0.30 Hz
GB 0
PC 1.00

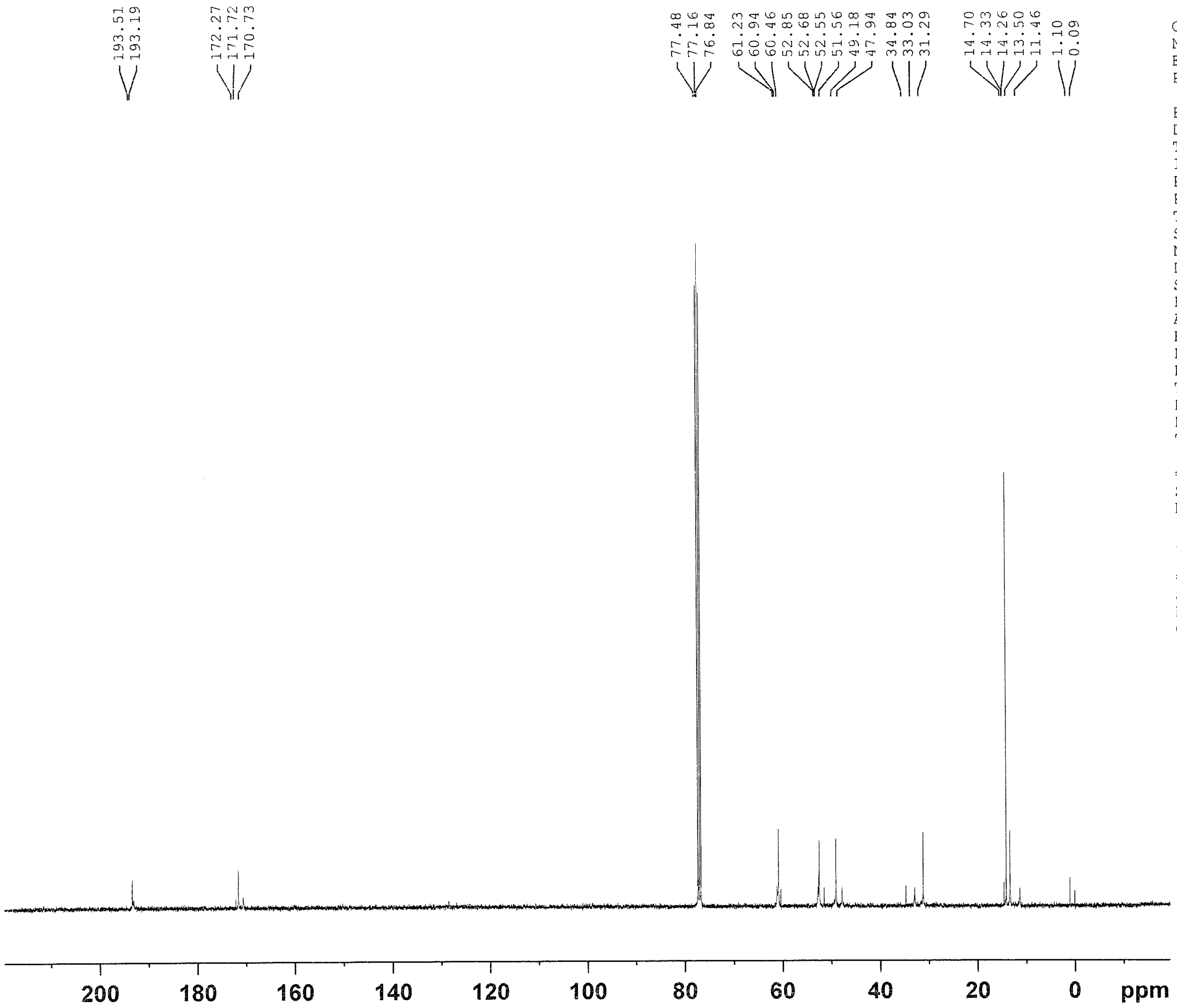


```
Current Data Parameters
NAME          SN0321-L1
EXPNO                11
PROCNO                1

F2 - Acquisition Parameters
Date_          20200821
Time              20.05
INSTRUM           spect
PROBHD   5 mm PABBO BB/
PULPROG         zgpg30
TD               65536
SOLVENT          CDC13
NS                1024
DS                   4
SWH          24038.461 Hz
FIDRES        0.366798 Hz
AQ           1.3631488 sec
RG               199.3
DW              20.800 usec
DE                6.50 usec
TE               295.0 K
D1          2.00000000 sec
D11         0.03000000 sec
TD0                  1

======== CHANNEL f1 ========
SFO1       100.5272687 MHz
NUC1               13C
P1                8.60 usec
PLW1       87.00000000 W

======== CHANNEL f2 ========
SFO2       399.7515990 MHz
NUC2                1H
CPDPRG[2       waltz16
PCPD2            90.00 usec
PLW2       19.20000076 W
PLW12       0.20000000 W
PLW13       0.15552001 W

F2 - Processing parameters
SI               32768
SF         100.5172100 MHz
WDW                 EM
SSB      0
LB                1.00 Hz
GB       0
PC                1.40
```

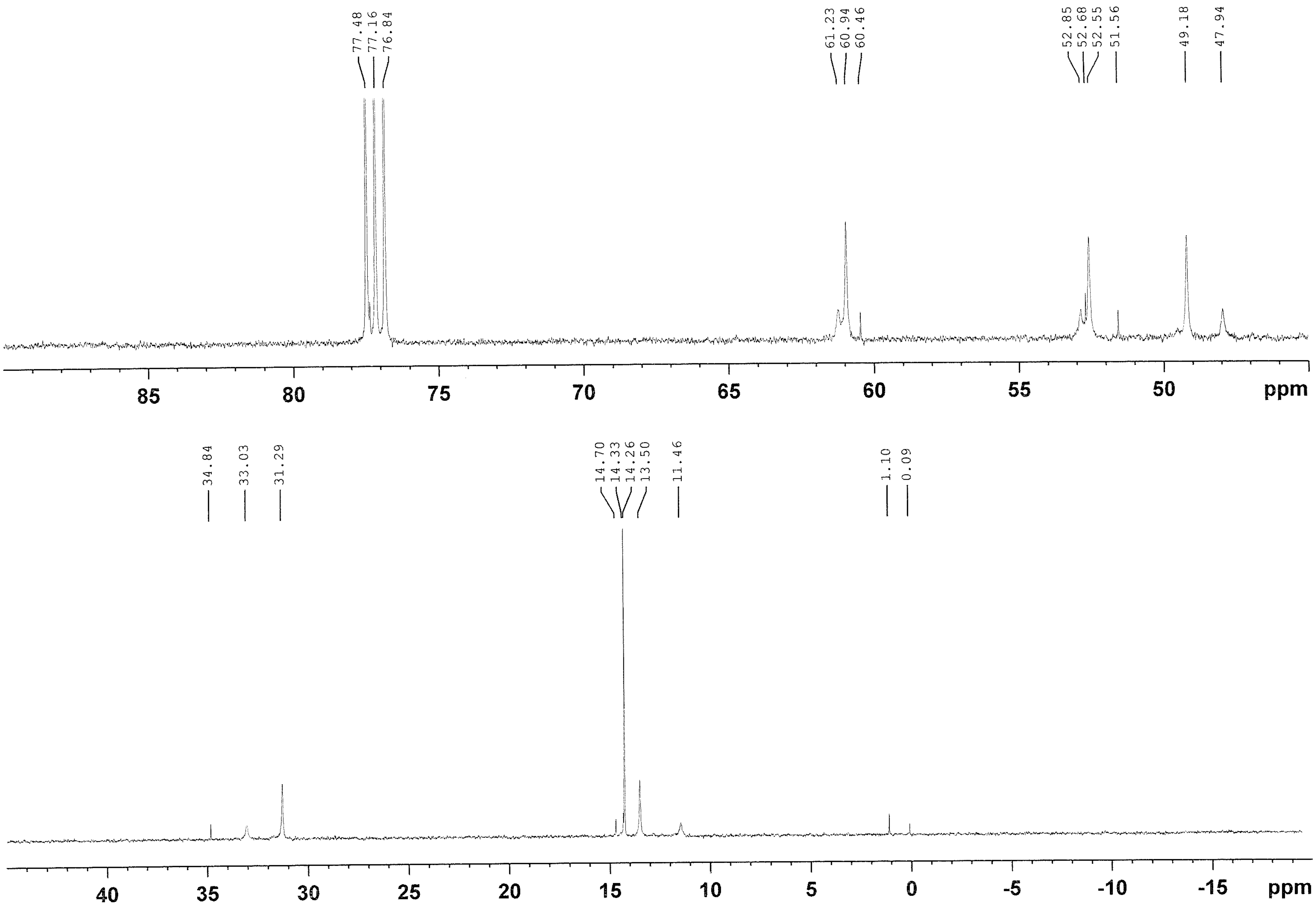
77.48
77.16
76.84
61.23
60.94
60.46
52.85
52.68
52.55
51.56
49.18
47.94
85
80
75
70
65
60
55
50
ppm
34.84
33.03
31.29
14.70
14.33
14.26
13.50
11.46
1.10
0.09
40
35
30
25
20
15
10
5
0
-5
-10
-15
ppm

SHIMADZU

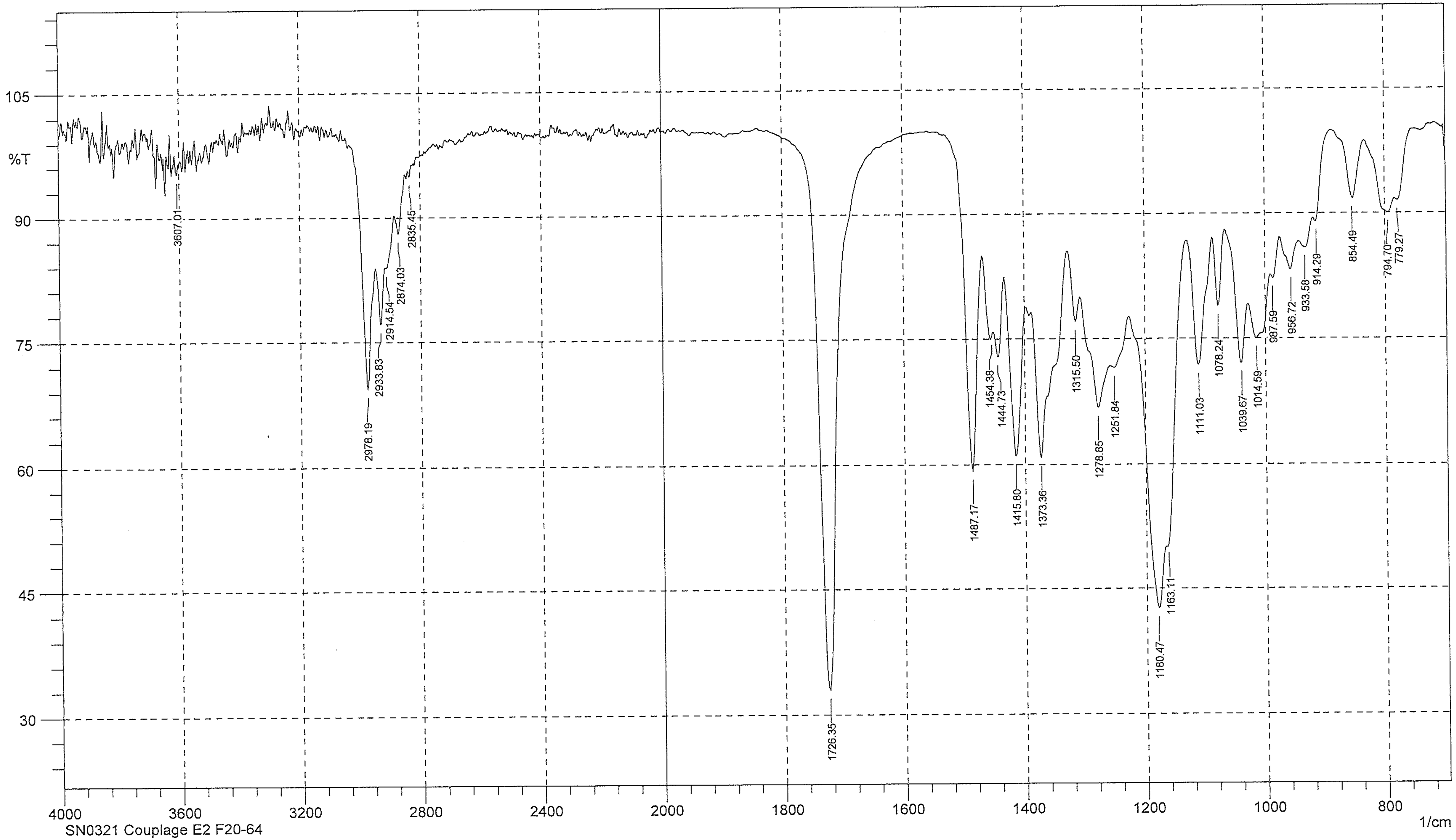

SN0321 Couplage E2 F20-64

Comment;
SN0321 Couplage E2 F20-64

No. of Scans;
Resolution;
Apodization;

Date/Time; 21/08/2020 09:14:03
User; Administrateur

Elemental Composition Report

Multiple Mass Analysis: 2 mass(es) processed

Tolerance = 5.0 PPM / DBE: min = 0.0, max = 7.0
Element prediction: Off
Number of isotope peaks used for i-FIT = 4

Monoisotopic Mass, Even Electron Ions
2538 formula(e) evaluated with 3 results within limits (all results (up to 1000) for each mass)
Elements Used:
C: 0-500 H: 0-1000 N: 0-3 O: 0-6 Na: 0-1 S: 0-4

2009016 78 (0.471) Cm (76:79-(4:14+406:411)) 02-Sep-2020

SN0321L1
1: TOF MS ES+ 2.64e+004

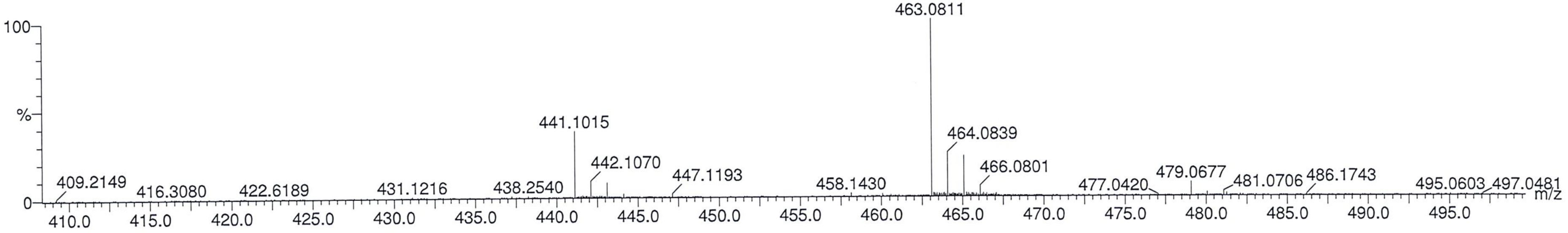

Mass	RA	Calc. Mass	mDa	PPM	DBE	i-FIT	i-FIT (Norm)	Formula
Minimum:	25.00				0.0			
Maximum:	100.00		5.0	5.0	7.0			
441.1015	37.13	441.1010	0.5	1.1	3.5	397.3	3.3	C16 H29 N2 O4 S4
		441.1026	-1.1	-2.5	4.5	394.1	0.0	C19 H30 O2 Na S4
463.0811	100.00	463.0830	-1.9	-4.1	3.5	576.2	0.0	C16 H28 N2 O4 Na S4

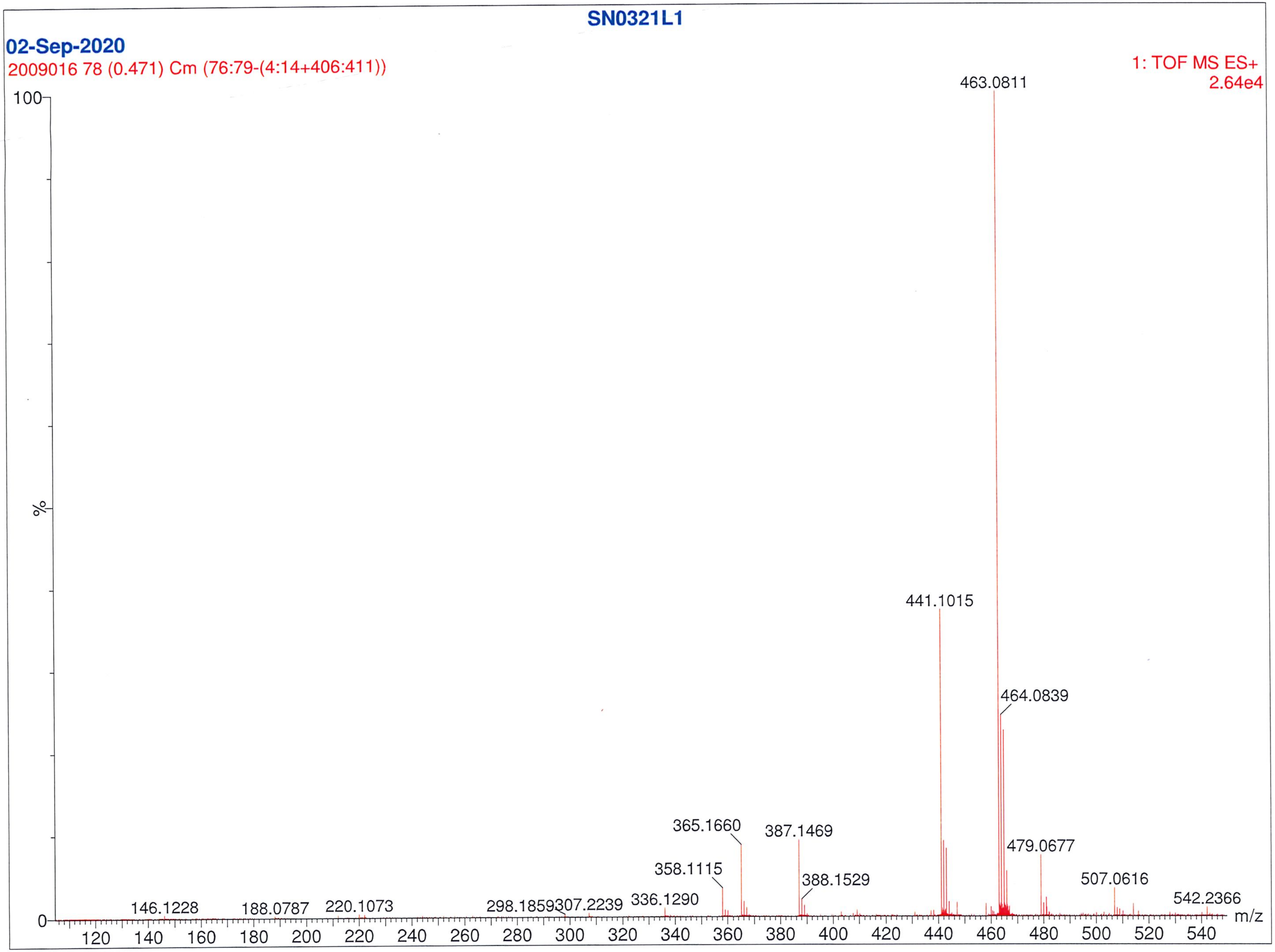
SN0321L1
02-Sep-2020
2009016 78 (0.471) Cm (76:79-(4:14+406:411))
1: TOF MS ES+
2.64e4
463.0811
441.1015
464.0839
365.1660
387.1469
479.0677
358.1115
388.1529
507.0616
542.2366
146.1228
188.0787
220.1073
298.1859
307.2239
336.1290
100
%
0
m/z
120
140
160
180
200
220
240
260
280
300
320
340
360
380
400
420
440
460
480
500
520
540

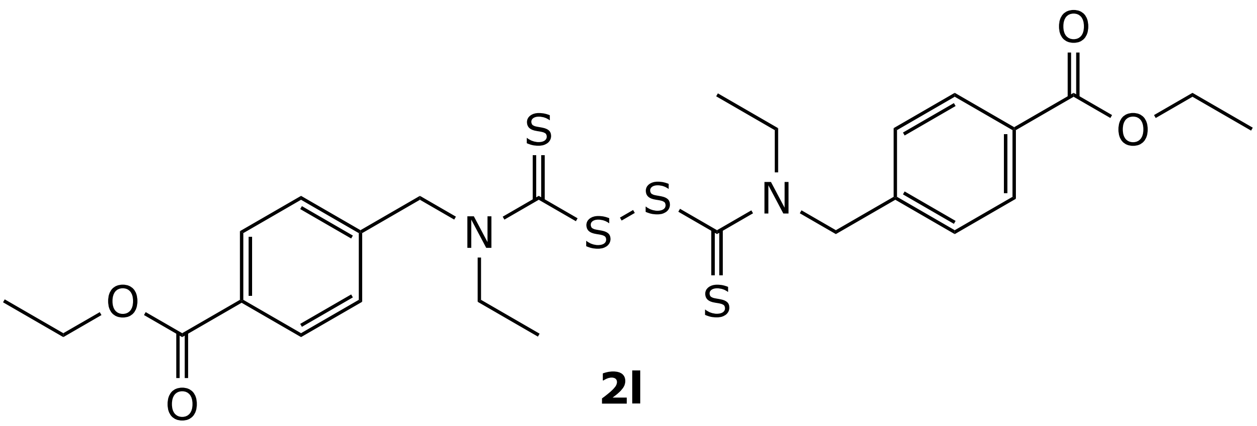

2l

Chemical Formula: $C_{26}H_{32}N_2O_4S_4$
Exact Mass: 564.1245

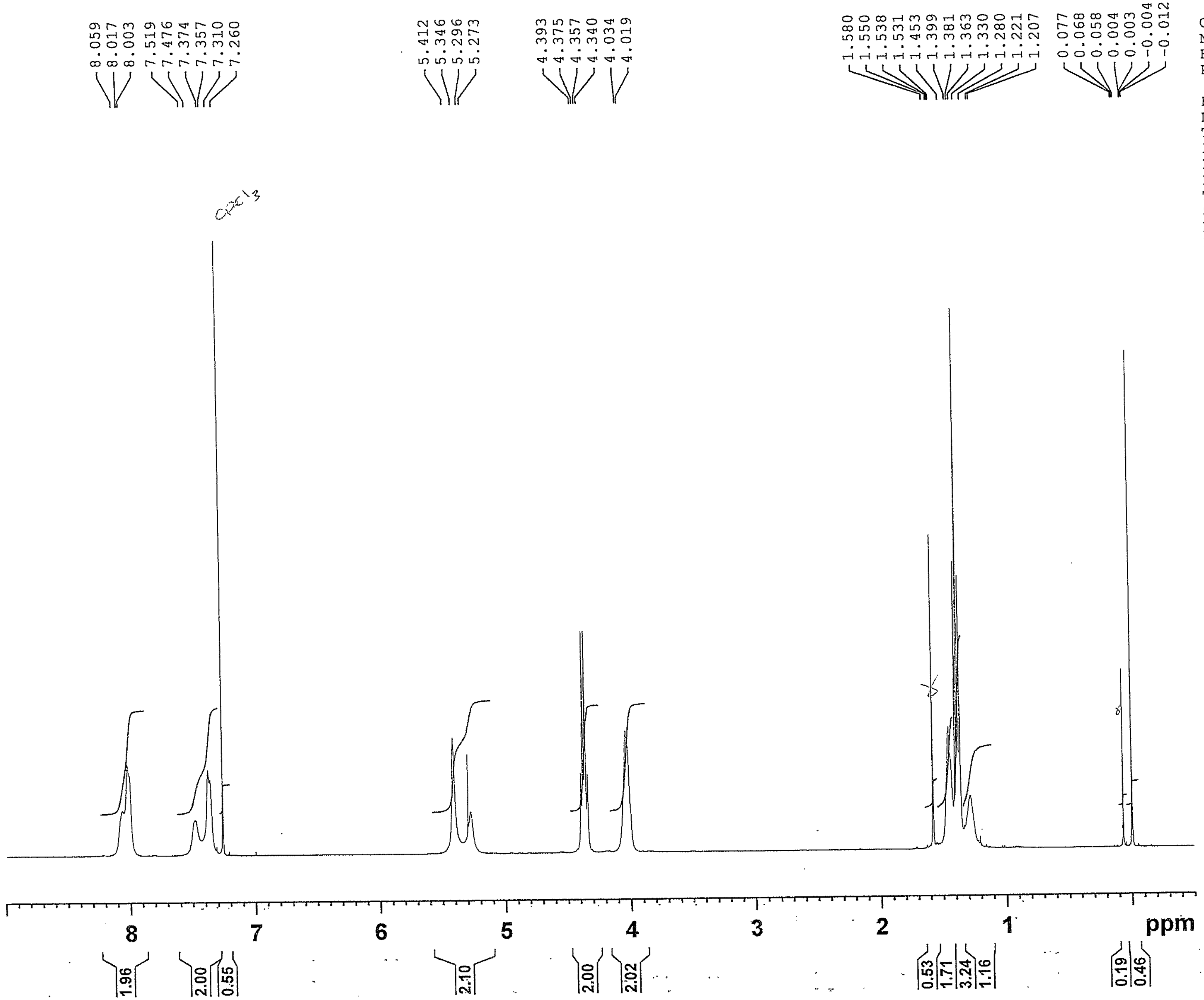

Current Data Parameters
NAME SN0317-L1
EXPNO 10
PROCNO 1

F2 - Acquisition Parameters
Date_ 20200706
Time 17.46
INSTRUM spect
PROBHD 5 mm PABBO BB/
PULPROG zg30
TD 32768
SOLVENT CDCl3
NS 16
DS 2
SWH 7978.724 Hz
FIDRES 0.243491 Hz
AQ 2.0534613 sec
RG 107.99
DW 62.667 usec
DE 6.50 usec
TE 296.2 K
D1 1.00000000 sec
TD0 1

======== CHANNEL f1 ========
SFO1 399.7524686 MHz
NUC1 1H
P1 9.00 usec
PLW1 19.20000076 W

F2 - Processing parameters
SI 65536
SF 399.7500097 MHz
WDW EM
SSB 0
LB 0.30 Hz
GB 0
PC 1.00

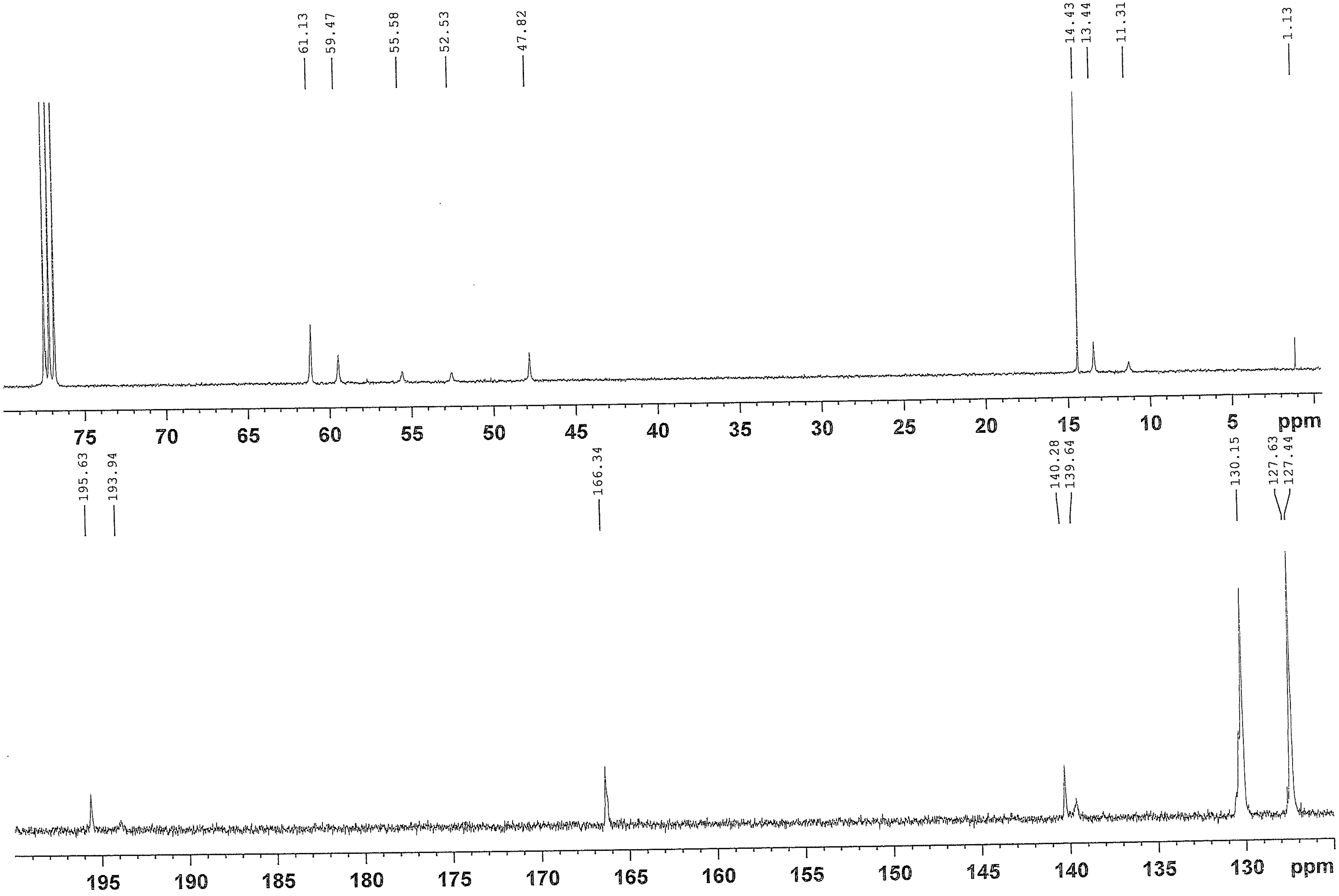
61.13
59.47
55.58
52.53
47.82
14.43
13.44
11.31
1.13
75
70
65
60
55
50
45
40
35
30
25
20
15
10
5
ppm
195.63
193.94
166.34
140.28
139.64
130.15
127.63
127.44
195
190
185
180
175
170
165
160
155
150
145
140
135
130
ppm

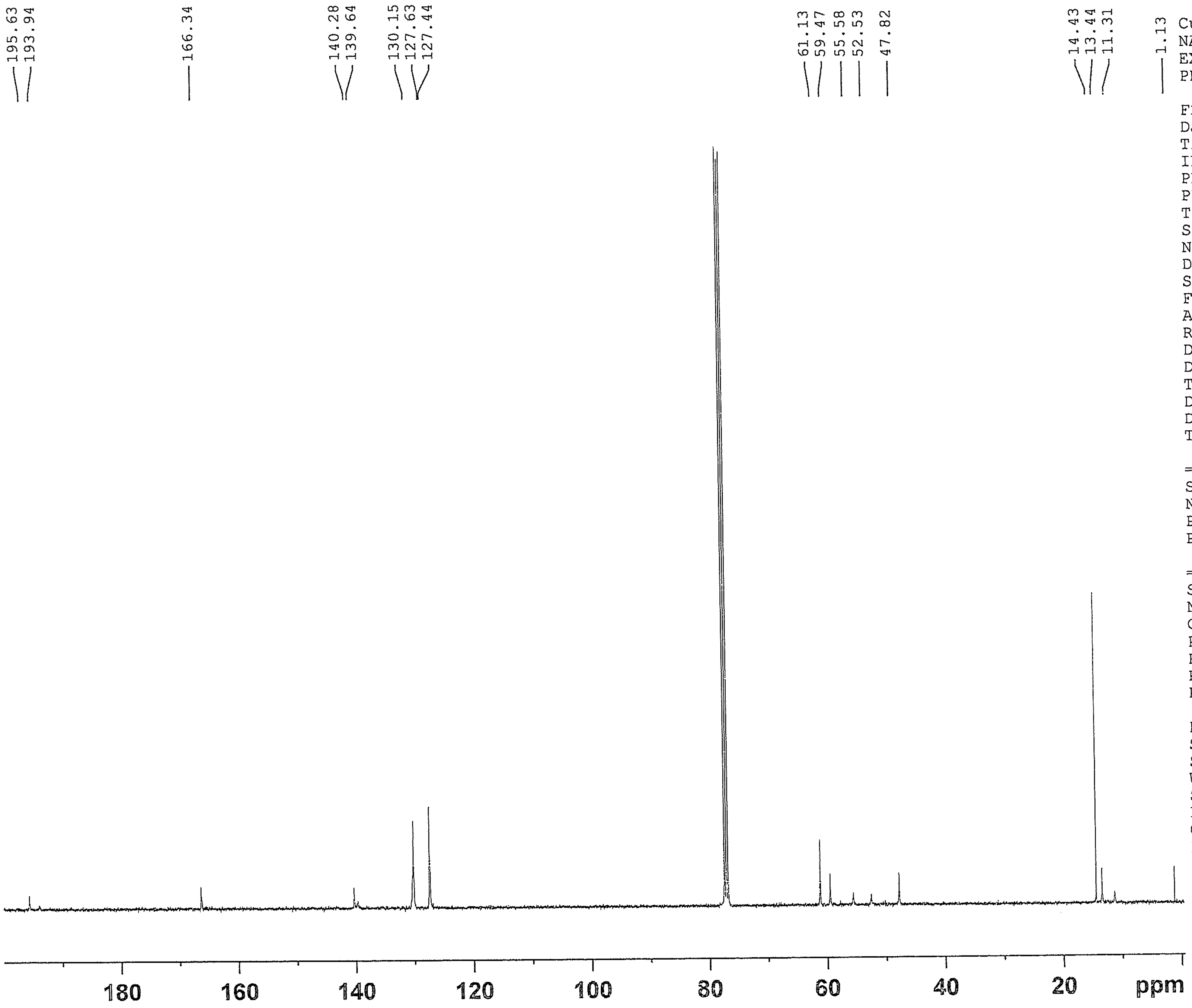

Current Data Parameters
NAME SN0317
EXPNO 20
PROCNO 1

F2 - Acquisition Parameters
Date_ 20200716
Time 23.05
INSTRUM spect
PROBHD 5 mm PABBO BB/
PULPROG zgpg30
TD 65536
SOLVENT CDCl3
NS 2048
DS 4
SWH 24038.461 Hz
FIDRES 0.366798 Hz
AQ 1.3631488 sec
RG 199.3
DW 20.800 usec
DE 6.50 usec
TE 295.0 K
D1 2.00000000 sec
D11 0.03000000 sec
TD0 1

======== CHANNEL f1 ========
SFO1 100.5272687 MHz
NUC1 13C
P1 8.60 usec
PLW1 87.00000000 W

======== CHANNEL f2 ========
SFO2 399.7515990 MHz
NUC2 1H
CPDPRG[2 waltz16
PCPD2 90.00 usec
PLW2 19.20000076 W
PLW12 0.20000000 W
PLW13 0.15552001 W

F2 - Processing parameters
SI 32768
SF 100.5172086 MHz
WDW EM
SSB 0
LB 1.00 Hz
GB 0
PC 1.40

SHIMADZU

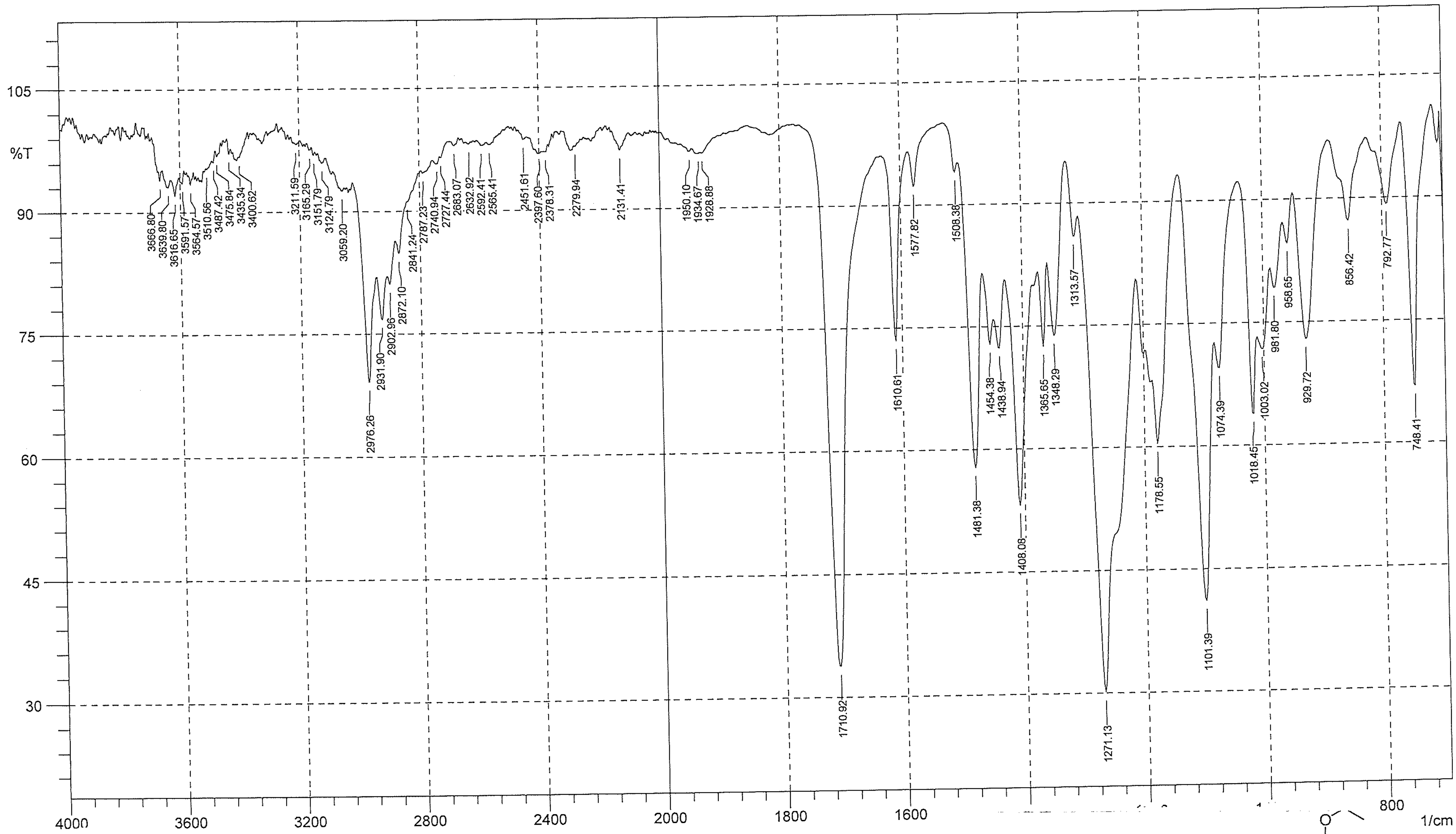

105
90
75
60
45
30
%T
4000
3600
3200
2800
2400
2000
1800
1600
800
1/cm
3666.80
3639.80
3616.65
3591.57
3564.57
3510.56
3487.42
3475.84
3435.34
3400.62
3211.59
3165.29
3151.79
3124.79
3059.20
2976.26
2931.90
2902.96
2872.10
2841.24
2787.23
2740.94
2727.44
2683.07
2632.92
2592.41
2565.41
2451.61
2397.60
2378.31
2279.94
2131.41
1950.10
1934.67
1928.88
1710.92
1610.61
1577.82
1508.38
1481.38
1454.38
1438.94
1408.08
1365.65
1348.29
1313.57
1271.13
1178.55
1101.39
1074.39
1018.45
1003.02
981.80
958.65
929.72
856.42
792.77
748.41

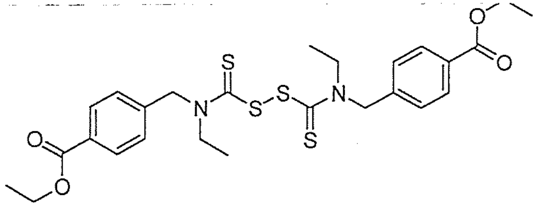

5

Elemental Composition Report

Single Mass Analysis

Tolerance = 10.0 PPM / DBE: min = 0.0, max = 15.0
Element prediction: Off
Number of isotope peaks used for i-FIT = 4

Monoisotopic Mass, Even Electron Ions
1096 formula(e) evaluated with 1 results within limits (all results (up to 1000) for each mass)
Elements Used:
C: 0-500 H: 0-1000 N: 0-5 O: 0-5 S: 0-4

SN0317L1
1: TOF MS ES+

2007172 297 (1.807) Cm (290:304) 17-Jul-2020

1.59e+004

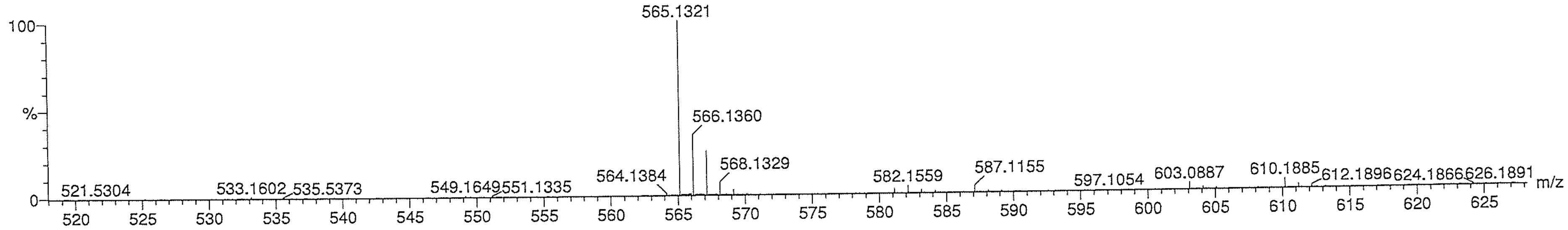

		mDa	PPM	DBE			
Minimum:				0.0			
Maximum:		5.0	10.0	15.0			

Mass	Calc. Mass	mDa	PPM	DBE	i-FIT	i-FIT (Norm)	Formula
565.1321	565.1323	-0.2	-0.4	11.5	543.8	0.0	C26 H33 N2 O4 S4
